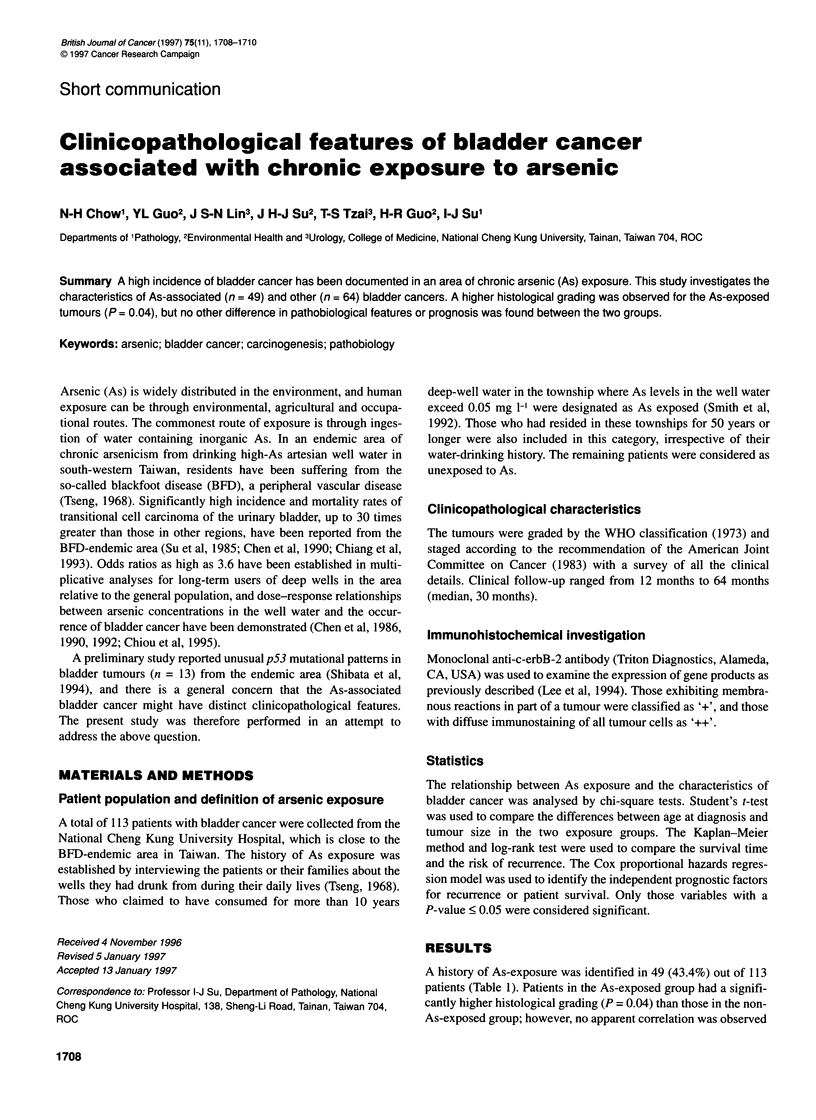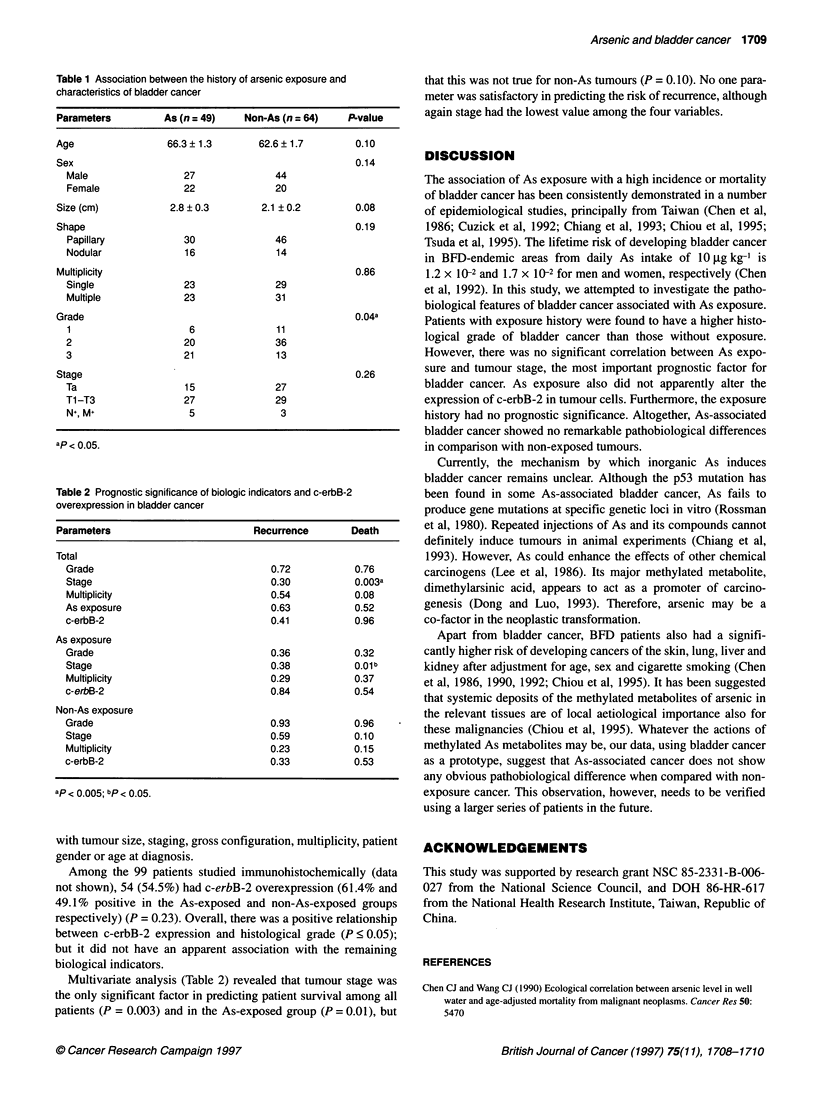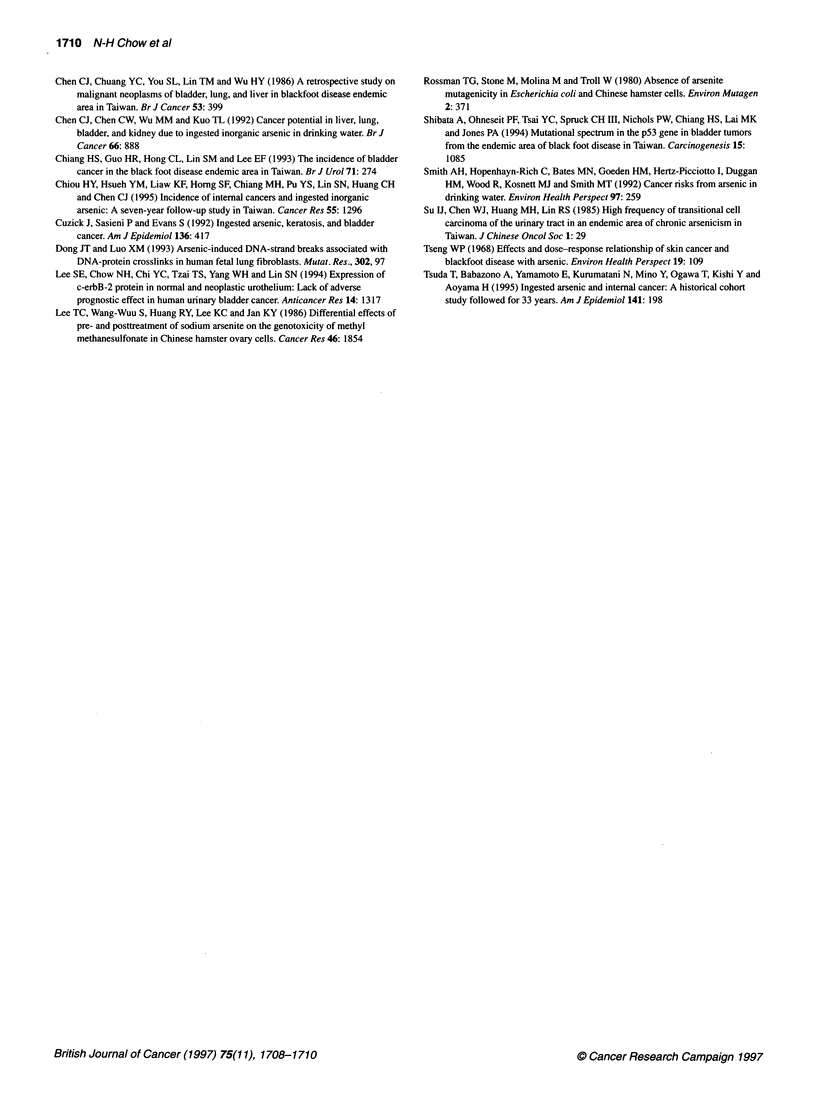# Clinicopathological features of bladder cancer associated with chronic exposure to arsenic.

**DOI:** 10.1038/bjc.1997.291

**Published:** 1997

**Authors:** N. H. Chow, Y. L. Guo, J. S. Lin, J. H. Su, T. S. Tzai, H. R. Guo, I. J. Su

**Affiliations:** Department of Pathology, College of Medicine, National Cheng Kung University, Tainan, Taiwan, ROC.

## Abstract

A high incidence of bladder cancer has been documented in an area of chronic arsenic (As) exposure. This study investigates the characteristics of As-associated (n = 49) and other (n = 64) bladder cancers. A higher histological grading was observed for the As-exposed tumours (P = 0.04), but no other difference in pathobiological features or prognosis was found between the two groups.


British Journal of Cancer (1997) 75(11), 1708–1710
© 1997 Cancer Research Campaign

Short communication

### Clinicopathological features of bladder cancer associated with chronic exposure to arsenic

**N-H Chow[1], YL Guo[2], J S-N Lin[3], J H-J Su[2], T-S Tzai[3], H-R Guo[2], I-J Su[1]**

Departments of [1]Pathology, [2]Environmental Health and [3]Urology, College of Medicine, National Cheng Kung University, Tainan, Taiwan 704, ROC

**Summary** A high incidence of bladder cancer has been documented in an area of chronic arsenic (As) exposure. This study investigates the characteristics of As-associated ($n = 49$) and other ($n = 64$) bladder cancers. A higher histological grading was observed for the As-exposed tumours ($P = 0.04$), but no other difference in pathobiological features or prognosis was found between the two groups.

**Keywords:** arsenic; bladder cancer; carcinogenesis; pathobiology

Arsenic (As) is widely distributed in the environment, and human exposure can be through environmental, agricultural and occupational routes. The commonest route of exposure is through ingestion of water containing inorganic As. In an endemic area of chronic arsenicism from drinking high-As artesian well water in south-western Taiwan, residents have been suffering from the so-called blackfoot disease (BFD), a peripheral vascular disease (Tseng, 1968). Significantly high incidence and mortality rates of transitional cell carcinoma of the urinary bladder, up to 30 times greater than those in other regions, have been reported from the BFD-endemic area (Su et al, 1985; Chen et al, 1990; Chiang et al, 1993). Odds ratios as high as 3.6 have been established in multiplicative analyses for long-term users of deep wells in the area relative to the general population, and dose–response relationships between arsenic concentrations in the well water and the occurrence of bladder cancer have been demonstrated (Chen et al, 1986, 1990, 1992; Chiou et al, 1995).

A preliminary study reported unusual *p53* mutational patterns in bladder tumours ($n = 13$) from the endemic area (Shibata et al, 1994), and there is a general concern that the As-associated bladder cancer might have distinct clinicopathological features. The present study was therefore performed in an attempt to address the above question.

### MATERIALS AND METHODS

### Patient population and definition of arsenic exposure

A total of 113 patients with bladder cancer were collected from the National Cheng Kung University Hospital, which is close to the BFD-endemic area in Taiwan. The history of As exposure was established by interviewing the patients or their families about the wells they had drunk from during their daily lives (Tseng, 1968). Those who claimed to have consumed for more than 10 years deep-well water in the township where As levels in the well water exceed 0.05 mg $l^{-1}$ were designated as As exposed (Smith et al, 1992). Those who had resided in these townships for 50 years or longer were also included in this category, irrespective of their water-drinking history. The remaining patients were considered as unexposed to As.

### Clinicopathological characteristics

The tumours were graded by the WHO classification (1973) and staged according to the recommendation of the American Joint Committee on Cancer (1983) with a survey of all the clinical details. Clinical follow-up ranged from 12 months to 64 months (median, 30 months).

### Immunohistochemical investigation

Monoclonal anti-c-erbB-2 antibody (Triton Diagnostics, Alameda, CA, USA) was used to examine the expression of gene products as previously described (Lee et al, 1994). Those exhibiting membranous reactions in part of a tumour were classified as '+', and those with diffuse immunostaining of all tumour cells as '++'.

### Statistics

The relationship between As exposure and the characteristics of bladder cancer was analysed by chi-square tests. Student's *t*-test was used to compare the differences between age at diagnosis and tumour size in the two exposure groups. The Kaplan–Meier method and log-rank test were used to compare the survival time and the risk of recurrence. The Cox proportional hazards regression model was used to identify the independent prognostic factors for recurrence or patient survival. Only those variables with a $P$-value $\leq 0.05$ were considered significant.

### RESULTS

A history of As-exposure was identified in 49 (43.4%) out of 113 patients (Table 1). Patients in the As-exposed group had a significantly higher histological grading ($P = 0.04$) than those in the non-As-exposed group; however, no apparent correlation was observed

*Received 4 November 1996*
*Revised 5 January 1997*
*Accepted 13 January 1997*

*Correspondence to:* Professor I-J Su, Department of Pathology, National Cheng Kung University Hospital, 138, Sheng-Li Road, Tainan, Taiwan 704, ROC

**Table 1** Association between the history of arsenic exposure and characteristics of bladder cancer

| Parameters | As ($n$ = 49) | Non-As ($n$ = 64) | $P$-value |
|---|---|---|---|
| Age | 66.3 ± 1.3 | 62.6 ± 1.7 | 0.10 |
| Sex | | | 0.14 |
| Male | 27 | 44 | |
| Female | 22 | 20 | |
| Size (cm) | 2.8 ± 0.3 | 2.1 ± 0.2 | 0.08 |
| Shape | | | 0.19 |
| Papillary | 30 | 46 | |
| Nodular | 16 | 14 | |
| Multiplicity | | | 0.86 |
| Single | 23 | 29 | |
| Multiple | 23 | 31 | |
| Grade | | | 0.04[a] |
| 1 | 6 | 11 | |
| 2 | 20 | 36 | |
| 3 | 21 | 13 | |
| Stage | | | 0.26 |
| Ta | 15 | 27 | |
| T1–T3 | 27 | 29 | |
| $N^+$, $M^+$ | 5 | 3 | |

[a]$P < 0.05$.

**Table 2** Prognostic significance of biologic indicators and c-erbB-2 overexpression in bladder cancer

| Parameters | Recurrence | Death |
|---|---|---|
| Total | | |
| Grade | 0.72 | 0.76 |
| Stage | 0.30 | 0.003[a] |
| Multiplicity | 0.54 | 0.08 |
| As exposure | 0.63 | 0.52 |
| c-erbB-2 | 0.41 | 0.96 |
| As exposure | | |
| Grade | 0.36 | 0.32 |
| Stage | 0.38 | 0.01[b] |
| Multiplicity | 0.29 | 0.37 |
| c-*erb*B-2 | 0.84 | 0.54 |
| Non-As exposure | | |
| Grade | 0.93 | 0.96 |
| Stage | 0.59 | 0.10 |
| Multiplicity | 0.23 | 0.15 |
| c-erbB-2 | 0.33 | 0.53 |

[a]$P < 0.005$; [b]$P < 0.05$.

with tumour size, staging, gross configuration, multiplicity, patient gender or age at diagnosis.

Among the 99 patients studied immunohistochemically (data not shown), 54 (54.5%) had c-*erb*B-2 overexpression (61.4% and 49.1% positive in the As-exposed and non-As-exposed groups respectively) ($P$ = 0.23). Overall, there was a positive relationship between c-erbB-2 expression and histological grade ($P \leq 0.05$); but it did not have an apparent association with the remaining biological indicators.

Multivariate analysis (Table 2) revealed that tumour stage was the only significant factor in predicting patient survival among all patients ($P$ = 0.003) and in the As-exposed group ($P$ = 0.01), but that this was not true for non-As tumours ($P$ = 0.10). No one parameter was satisfactory in predicting the risk of recurrence, although again stage had the lowest value among the four variables.

### DISCUSSION

The association of As exposure with a high incidence or mortality of bladder cancer has been consistently demonstrated in a number of epidemiological studies, principally from Taiwan (Chen et al, 1986; Cuzick et al, 1992; Chiang et al, 1993; Chiou et al, 1995; Tsuda et al, 1995). The lifetime risk of developing bladder cancer in BFD-endemic areas from daily As intake of 10 $\mu g\ kg^{-1}$ is $1.2 \times 10^{-2}$ and $1.7 \times 10^{-2}$ for men and women, respectively (Chen et al, 1992). In this study, we attempted to investigate the pathobiological features of bladder cancer associated with As exposure. Patients with exposure history were found to have a higher histological grade of bladder cancer than those without exposure. However, there was no significant correlation between As exposure and tumour stage, the most important prognostic factor for bladder cancer. As exposure also did not apparently alter the expression of c-erbB-2 in tumour cells. Furthermore, the exposure history had no prognostic significance. Altogether, As-associated bladder cancer showed no remarkable pathobiological differences in comparison with non-exposed tumours.

Currently, the mechanism by which inorganic As induces bladder cancer remains unclear. Although the p53 mutation has been found in some As-associated bladder cancer, As fails to produce gene mutations at specific genetic loci in vitro (Rossman et al, 1980). Repeated injections of As and its compounds cannot definitely induce tumours in animal experiments (Chiang et al, 1993). However, As could enhance the effects of other chemical carcinogens (Lee et al, 1986). Its major methylated metabolite, dimethylarsinic acid, appears to act as a promoter of carcinogenesis (Dong and Luo, 1993). Therefore, arsenic may be a co-factor in the neoplastic transformation.

Apart from bladder cancer, BFD patients also had a significantly higher risk of developing cancers of the skin, lung, liver and kidney after adjustment for age, sex and cigarette smoking (Chen et al, 1986, 1990, 1992; Chiou et al, 1995). It has been suggested that systemic deposits of the methylated metabolites of arsenic in the relevant tissues are of local aetiological importance also for these malignancies (Chiou et al, 1995). Whatever the actions of methylated As metabolites may be, our data, using bladder cancer as a prototype, suggest that As-associated cancer does not show any obvious pathobiological difference when compared with non-exposure cancer. This observation, however, needs to be verified using a larger series of patients in the future.

### ACKNOWLEDGEMENTS

This study was supported by research grant NSC 85-2331-B-006-027 from the National Science Council, and DOH 86-HR-617 from the National Health Research Institute, Taiwan, Republic of China.

### REFERENCES

Chen CJ and Wang CJ (1990) Ecological correlation between arsenic level in well water and age-adjusted mortality from malignant neoplasms. *Cancer Res* **50**: 5470

Chen CJ, Chuang YC, You SL, Lin TM and Wu HY (1986) A retrospective study on malignant neoplasms of bladder, lung, and liver in blackfoot disease endemic area in Taiwan. *Br J Cancer* **53**: 399

Chen CJ, Chen CW, Wu MM and Kuo TL (1992) Cancer potential in liver, lung, bladder, and kidney due to ingested inorganic arsenic in drinking water. *Br J Cancer* **66**: 888

Chiang HS, Guo HR, Hong CL, Lin SM and Lee EF (1993) The incidence of bladder cancer in the black foot disease endemic area in Taiwan. *Br J Urol* **71**: 274

Chiou HY, Hsueh YM, Liaw KF, Horng SF, Chiang MH, Pu YS, Lin SN, Huang CH and Chen CJ (1995) Incidence of internal cancers and ingested inorganic arsenic: A seven-year follow-up study in Taiwan. *Cancer Res* **55**: 1296

Cuzick J, Sasieni P and Evans S (1992) Ingested arsenic, keratosis, and bladder cancer. *Am J Epidemiol* **136**: 417

Dong JT and Luo XM (1993) Arsenic-induced DNA-strand breaks associated with DNA-protein crosslinks in human fetal lung fibroblasts. *Mutat. Res*., **302**, 97

Lee SE, Chow NH, Chi YC, Tzai TS, Yang WH and Lin SN (1994) Expression of c-erbB-2 protein in normal and neoplastic urothelium: Lack of adverse prognostic effect in human urinary bladder cancer. *Anticancer Res* **14**: 1317

Lee TC, Wang-Wuu S, Huang RY, Lee KC and Jan KY (1986) Differential effects of pre- and posttreatment of sodium arsenite on the genotoxicity of methyl methanesulfonate in Chinese hamster ovary cells. *Cancer Res* **46**: 1854

Rossman TG, Stone M, Molina M and Troll W (1980) Absence of arsenite mutagenicity in *Escherichia coli* and Chinese hamster cells. *Environ Mutagen* **2**: 371

Shibata A, Ohneseit PF, Tsai YC, Spruck CH III, Nichols PW, Chiang HS, Lai MK and Jones PA (1994) Mutational spectrum in the p53 gene in bladder tumors from the endemic area of black foot disease in Taiwan. *Carcinogenesis* **15**: 1085

Smith AH, Hopenhayn-Rich C, Bates MN, Goeden HM, Hertz-Picciotto I, Duggan HM, Wood R, Kosnett MJ and Smith MT (1992) Cancer risks from arsenic in drinking water. *Environ Health Perspect* **97**: 259

Su IJ, Chen WJ, Huang MH, Lin RS (1985) High frequency of transitional cell carcinoma of the urinary tract in an endemic area of chronic arsenicism in Taiwan. *J Chinese Oncol Soc* **1**: 29

Tseng WP (1968) Effects and dose–response relationship of skin cancer and blackfoot disease with arsenic. *Environ Health Perspect* **19**: 109

Tsuda T, Babazono A, Yamamoto E, Kurumatani N, Mino Y, Ogawa T, Kishi Y and Aoyama H (1995) Ingested arsenic and internal cancer: A historical cohort study followed for 33 years. *Am J Epidemiol* **141**: 198

© Cancer Research Campaign 1997